# An insight on the powerful of bacterial quorum sensing inhibition

**DOI:** 10.1007/s10096-024-04920-w

**Published:** 2024-08-19

**Authors:** Nourhan G. Naga, Mona I. Shaaban, Mohammad Magdy El-Metwally

**Affiliations:** 1 Botany and Microbiology Department, Faculty of Science, Alexandria University, Alexandria, Egypt; 2 Microbiology and Immunology Department, Faculty of Pharmacy, Mansoura University, Mansoura, Egypt; 3 Botany and Microbiology Department, Faculty of Science, Damanhour University, El-Behera, Egypt

**Keywords:** Quorum sensing, Biofilm formation, Quorum quenching enzymes, Phage therapy, Virulence factors

## Abstract

Bacteria have their own language through which they communicate with one another like all higher organisms. So, many researchers are working hard to identify and comprehend the components of this bacterial communication, known as quorum sensing (QS). In quorum sensing, bacteria use signaling molecules called autoinducers (AIs) to exchange information. Many natural compounds and extraction techniques have been intensively studied to disrupt bacterial signaling and examine their effectiveness for bacterial pathogenesis control. Quorum sensing inhibitors can interfere with QS and block the action of AI signaling molecules. Recent research indicates that quorum sensing inhibitors (QSIs) and quorum quenching enzymes (QQEs) show great promise in reducing the pathogenicity of bacteria and inhibiting biofilm synthesis. In addition, the effectiveness of QQEs and QSIs in experimental animal models was demonstrated. These are taken into account in the development of innovative medical devices, such as dressings and catheters, to prevent bacterial infections. The present review highlights this aspect with a prospective vision for its development and application.

## Introduction

**Fig. 1:**
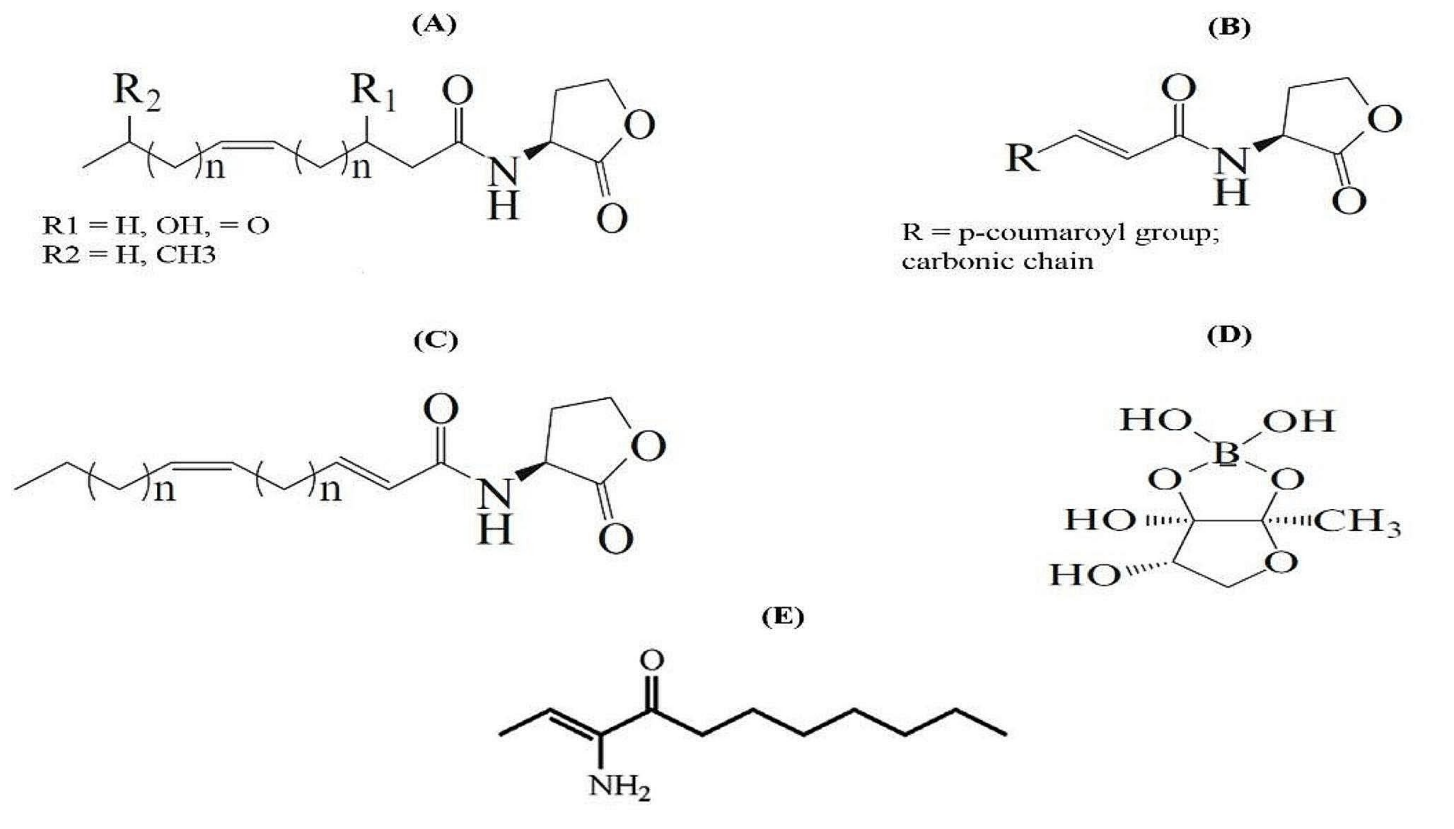
Some structures of bacterial autoinducers. acyl-homoserine lactone group AHLs (A, B, C), autoinducer-2 (AI-2, furanosyl borate diester) (D), and (*S*)-3-hydroxytridecan-4-one; CAI-1 (E)

Quorum sensing (QS) is the process of bacterial cell-to-cell communication that utilizes their activities to act together in a group manner [1]. At high cell density, on reaching the threshold level, QS allows bacteria to perform processes like biofilm formation, virulence factor synthesis, siderophore production, and enzyme production [2]. QS is characterized by the production of small signaling molecules called autoinducers (AIs), which are coordinated by cell density [3]. In Gram-positive bacteria such as *Staphylococcus* spp., autoinducing peptides (AIPs) have been shown to promote QS [4]. While acyl-homoserine lactones (AHLs) were found to be the AIs in Gram-negative bacteria such as *Acinetobacter* spp., *Pseudomonas* spp., and *Burkholderia* spp. AHLs are made up of an acyl chain and a lactone ring that varies in structure, length, and cellular activity Fig. 1 [5].

**Fig. 2:**
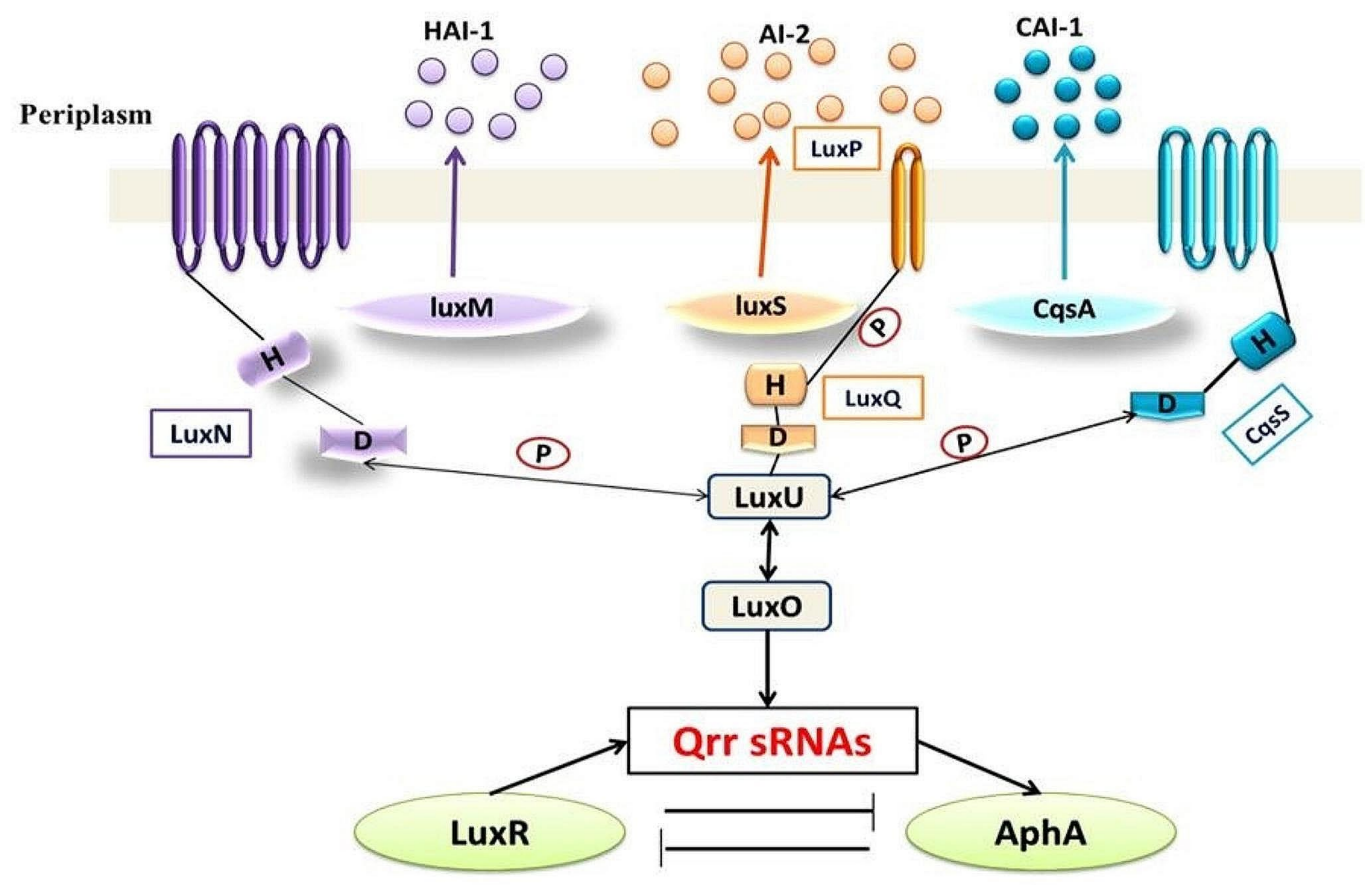
Quorum sensing cascade in *Vibrio harveyi*

**Fig. 3:**
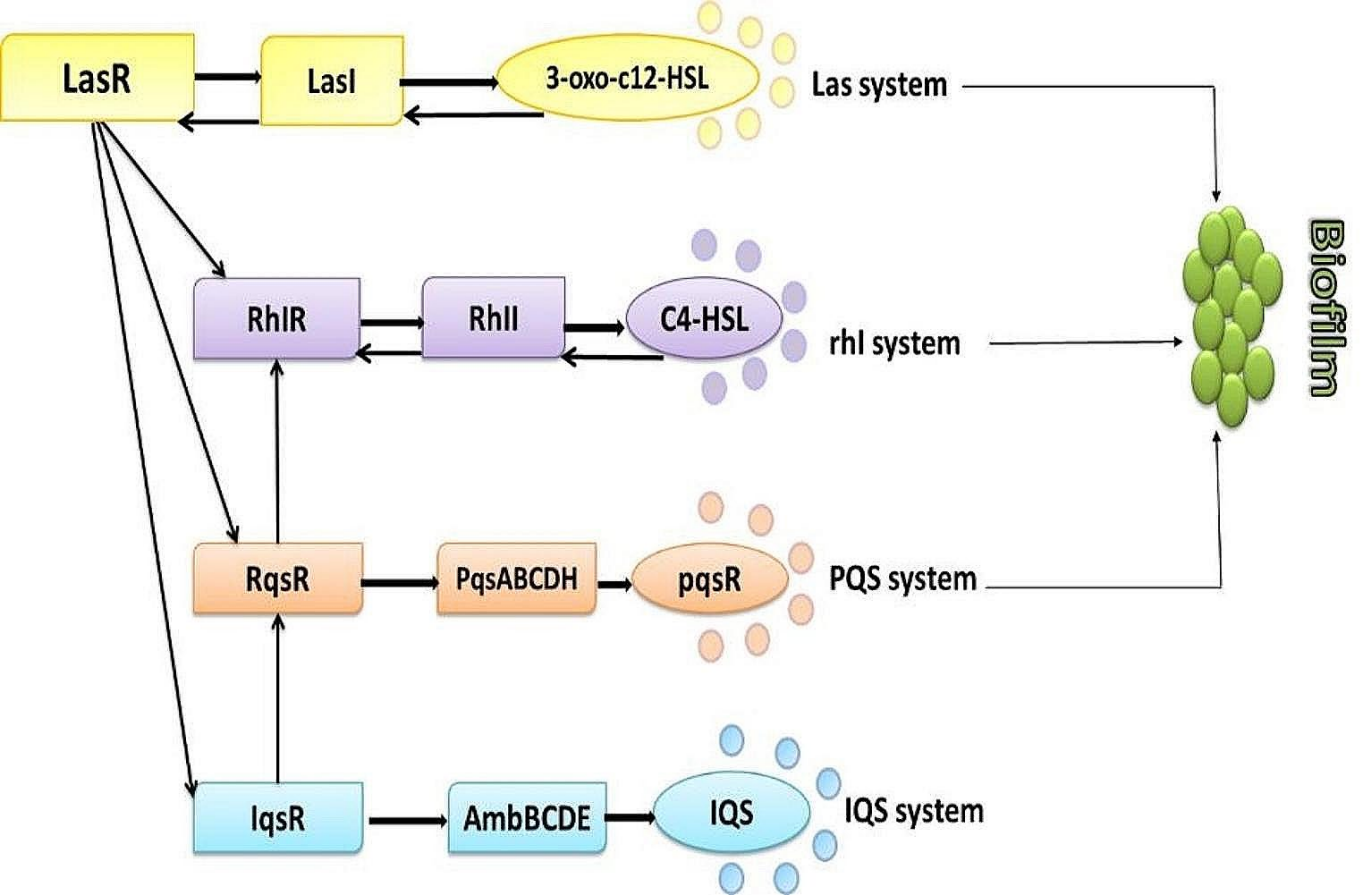
Quorum sensing cascade in *p. aeruginosa*

There are many other AIs that have also been identified, such as ketones which are used by *Legionella* spp. and *Vibrio cholera* spp. [6] and fatty acids that are utilized by *Burkholderia* spp. and *Xanthomonas* spp [7]. Autoinducer-2 (AI-2) is utilized by Gram-positive and Gram-negative bacteria Fig. 1 [8]. Some Gram-negative bacteria use multiple QS systems, either in parallel, like in *Vibrio harveyi*, where three systems are linked together to form a single regulatory cascade Fig. 2. The first system synthase is LuxM, which produces the AI; MAI-1, the second is LuxS, which produces AI-2, and the third is CqsA, which produces CAI-1. Others, such as *P. aeruginosa*, employ a hierarchical cascade with four QS systems. The first system is named LasI/LasR, the second is RhlI/RhlR, the third is PqsABCDE/PqsR, and the fourth is AmbBCDE/IqsR system [9, 10]. Each system employs a different AI; 3-oxododecanoyl-L-homoserine lactone (3-oxo-C12-HSL), N-butanoyl homoserine lactone (C4-HSL), 2-heptyl-3-hydroxy-4-quinolone (*Pseudomonas* quinolone signal, PQS), and 2-(2-hydroxyphenyl)-thiazole-4-carbaldehyde, respectively Fig. 3.

## Elimination of quorum sensing

There are a number of methods that can disrupt or quench bacterial communication, including: (i) using quorum sensing inhibitors (QSIs) for the interrupting of AIs [3, 11, 12] (ii) AIs scavenging by using macromolecules, antibodies of quorum quenching [13], or (iii) using QQ enzymes (QQEs) to hydrolyze the AIs extracellularly [14]. There are numerous natural compounds that can act as QSIs. For instance, polyphenols that were isolated from beans, carrots, chamomile [15], and *Trigonella stellata* [16]. Some other QSIs may be synthetic, such as aspirin [17], β-lactams [18], azithromycin [19], and benzothiazole derivatives[20].

**Fig. 4:**
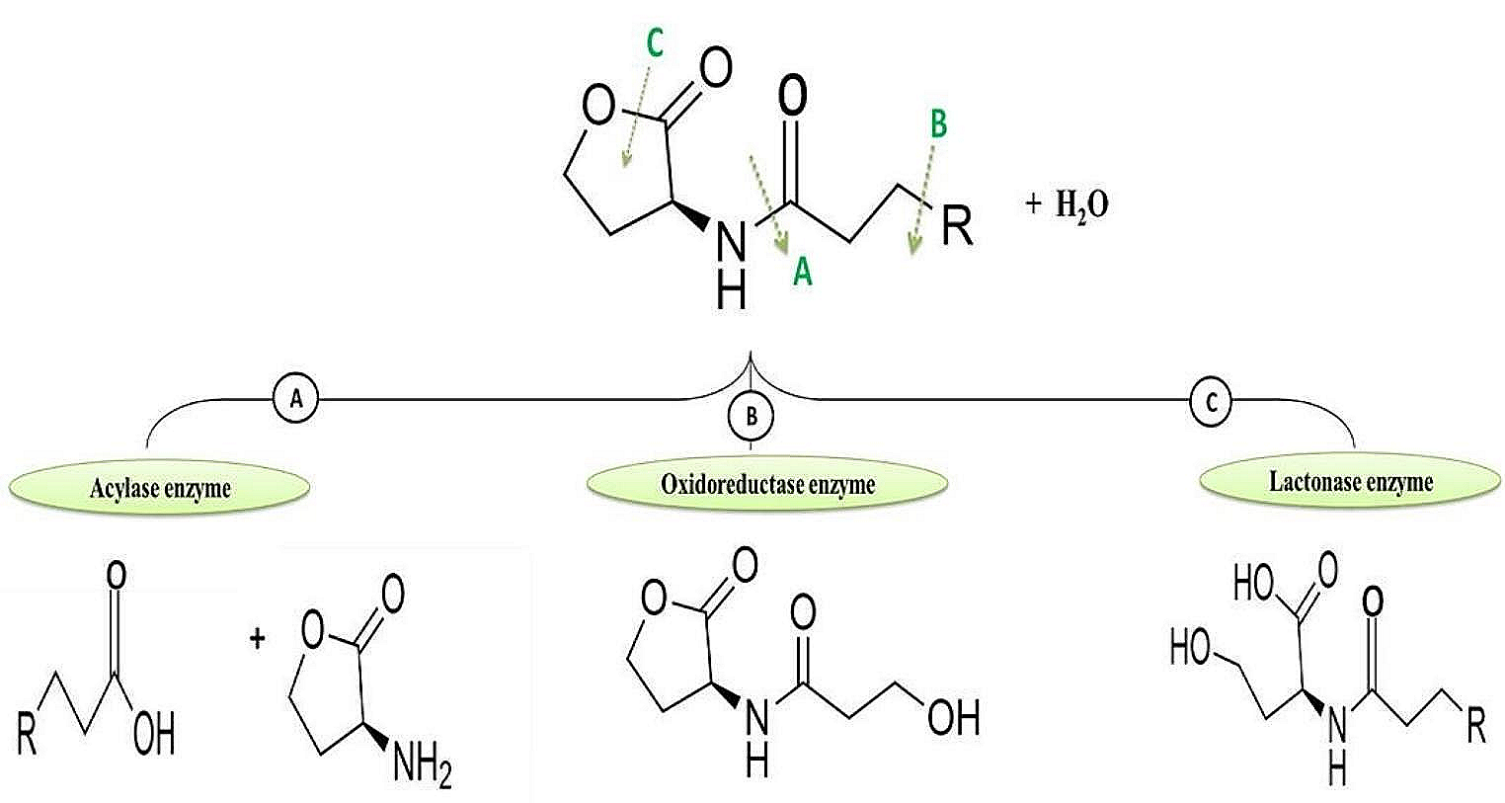
Enzymatic inactivation of AIs by acylase, oxidoreductase, and lactonase enzymes

Many QQEs were reported to disrupt QS in Gram-negative bacteria and degrade AHL. For example, the acylase enzyme breaks down the amide linkage between the side chain of fatty acid and lactone ring [21]. The acylase enzyme was isolated from *Ochrobactrum* sp. [22], *P. aeruginosa* [23], and *Streptomyces* sp. [24]. Also, lactonase enzymes that hydrolyze the lactone ring were isolated from *Bacillus* sp. [25], *Klebsiella pneumoniae*,* Arthrobacter sp.* [26], and *Rhodococcus erythropolis* [27]. The third family of known AHL QQEs are the oxidoreductases. This group of enzymes acts by oxidizing or reducing the acyl chains of the AHLs rather than destroying them [3]. These processes do not result in signal degradation, but they modify the specificity, which has an impact on the interaction between the signal and the receptor Fig. 4. Paraoxonases enzymes (PONs) are another group of human enzymes that have recently been identified as QQ agents. PONs are capable of AHL-mediated QS disruption as acylase and lactonase enzymes [28]. PONs are thought to play a significant role in innate human defense mechanisms that inactivate harmful *P. aeruginosa* AHL signals [29].

QQ approaches have applications in a variety of fields like agriculture, water treatment, fisheries and aquaculture, and health care [3, 30]. Recently, considerable attention has been paid to finding novel therapeutic approaches with the increasing antibiotic resistance [31, 32].

## The relationship between antimicrobial resistance and QS

**Fig. 5:**
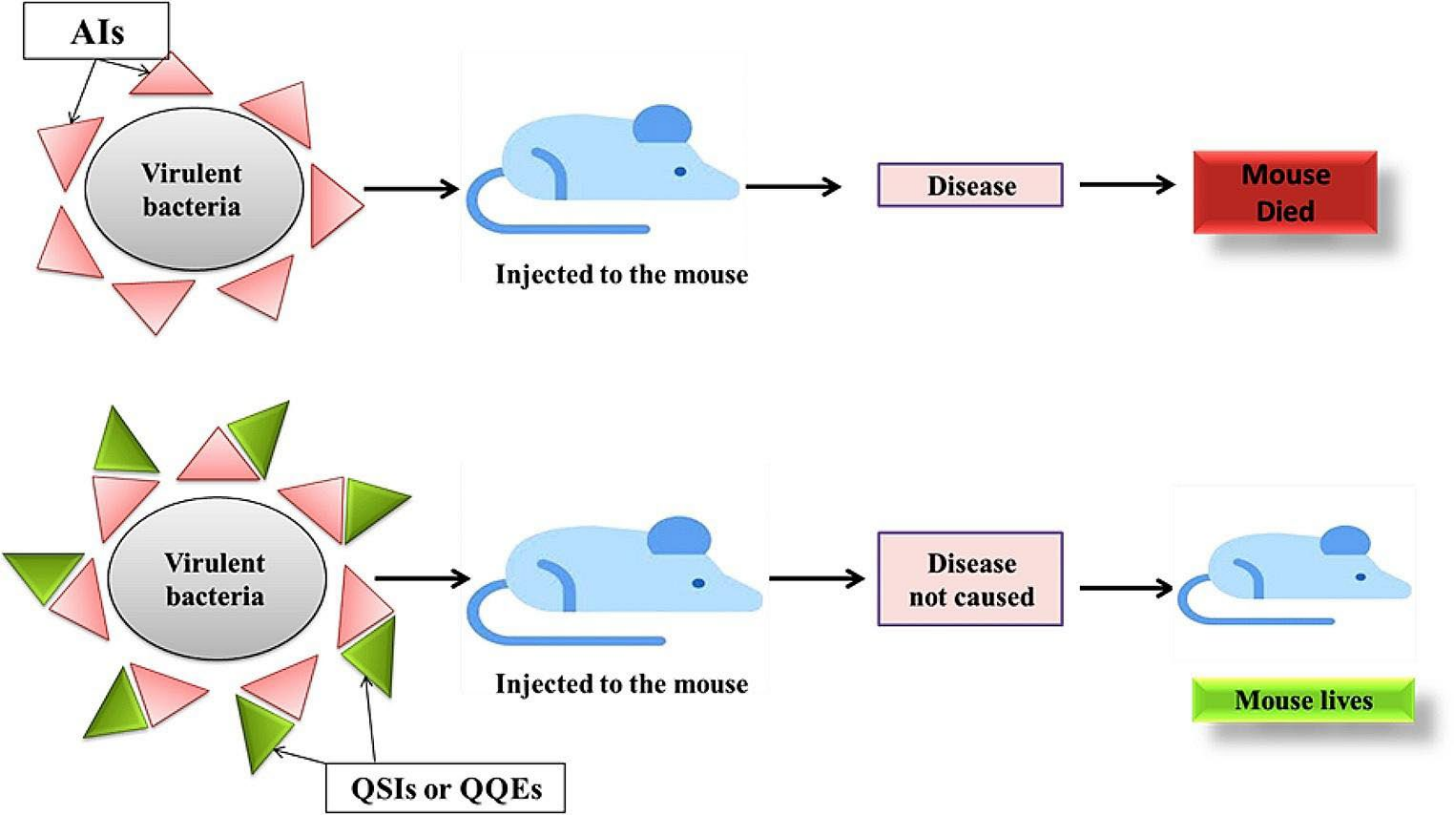
The effect of QSIs and QQEs applications on the mice’s life

Several studies reported that there is a correlation between QS and antibiotic resistance because QS is a crucial regulator of biofilm development. As an instance, the incorporation of AHLs into a culture of *P. aeruginosa* increased resistance against ciprofloxacin and carbenicillin antibiotics [33]. Also, a transcriptomic study in *P. aeruginosa* PA14 showed that QS increased PqsR expression, which protects against β-lactam antibiotics and H_2_O_2_ [34]. The global regulator VqsM, which promotes QS, was identified as a major contributor to antibiotic resistance against quinolones, kanamycin, and tetracycline [35]. The physiological factors play a role in QS-mediated antibiotic tolerance, but many studies have emphasized the significance of biofilm in bacterial antibiotic resistance [36]. Biofilm is a heterogeneous multicellular structure attached to a solid substrate [37]. The extracellular matrix (ECM) of biofilms, composed of extracellular DNA, polysaccharides, and proteins that plays a crucial role in enhancing antibiotic resistance. This resistance is attributed to the protective ECM barrier, slower bacterial growth rates within the biofilm, and the presence of persisted cells [38]. This biofilm also can significantly enhance the bacterial pathogenicity through several mechanisms like enhancing the bacterial communication and coordination via QS, this allows bacteria to coordinate their behavior, including the regulation of virulence factor production [39, 40]. They protect bacteria from immune responses by acting as a physical barrier, preventing phagocytosis and neutralizing antibodies [41]. The close proximity of bacterial cells within a biofilm facilitates horizontal gene transfer, spreading antibiotic resistance genes and virulence factors, thereby increasing pathogenic potential [42]. Biofilms contribute to persistent infections, as they are difficult to eradicate and can lead to chronic and recurrent infections. Effective communication through quorum sensing within biofilms regulates virulence genes and coordinates toxin release [43]. Additionally, Biofilms provide a stable environment for bacteria, enabling survival under hostile conditions such as nutrient deprivation and pH changes [44]. As a result, bacteria within a biofilm can mount a more coordinated and effective attack on the host. Some QSIs were employed to increase the sensitivity of antibiotics and to lower the doses of antibiotics [45]. Additionally, in the mouse model, combining antimicrobial medications with QQEs as lactonase had a synergistic impact and boosted antibiotic efficiency (Fig. 5). The effectiveness of QQEs and QSIs in the treatment and prevention of infections has been approved. However, it cannot be used in alone to treat acute infections caused by antibiotic-resistant bacteria.

## Quorum sensing and biofilm formation

Biofilm-forming bacteria are believed to be 1000 times more resistant to antimicrobial agents [46] and it is estimated that biofilm-associated infections account for 80%. This occurs either through infected tissues, as in cystic fibrosis, or via contaminated devices. Biofilm encourages the emergence of various phenotypes and defense mechanisms, including alterations in cellular physiological state, physical barrier formation, and gene expression. Inhibitor that disrupts QS systems will consequently alter the biofilm formation. For instance, *P. aeruginosa*’s Las, Rhl, and PQS QS systems are necessary for the synthesis of biofilms (Fig. 3), and any changes are associated with increased susceptibility to both antimicrobials and the human immune system [47]. Some antibiotics like levofloxacin and meropenem were found to increase the expression of efflux pumps, which boost the production of AHL in *Acinetobacter baumannii* clinical isolates. As a result, biofilm development increases the antibiotic resistance. As mentioned earlier, QS controls both biofilm development and antibiotic resistance. So, the use of QQ molecules was studied. Using some pharmacological agents such as benzamide-benzimidazole in *P. aeruginosa* could inhibit the QS regulators, reduced biofilm development, and decreased antibiotic resistance [48]. Some QSIs, such as hamamelitannin and baicalin hydrate, improved the biofilm disintegration. In vivo and in vitro, they exhibited synergistic effects against Gram-negative bacteria when QSIs were coupled with antibiotics such as tobramycin, vancomycin, or clindamycin [49]. Besides, the efficacy of a wide range of antibiotics, such as quinolones [50], cephalosporins, and glycopeptides [48] was improved when added to QSIs.

### The relationship between sensitivity to bacteriophages and quorum sensing

In the last decade, there has been massive interest in using phage therapy to treat infections caused by multidrug-resistant (MDR) bacteria. Bacteriophages are the most numerous bacterial predators on Earth and are utilized for treating bacterial infections [51]. Despite the intriguing potential of bacteriophages as a solution, bacteria have developed mechanisms of resistance to evade their effects [52]. For example, bacteria can reduce phage entry by increasing extracellular matrix formation and altering the structure of phage receptors [53]. Bacteria can also recognize and degrade phage DNA with the aid of restriction enzymes [54]. The first pathogen demonstrating a correlation between bacteriophage sensitivity and quorum sensing (QS) was *P. aeruginosa* [55]. However, it took years to establish that QS modulates mechanisms of phage defense in *Escherichia coli* by reducing the chi adsorption rate and phage lambda. The absence of AI synthase genes in *Vibrio cholera* lowered phage resistance, which was restored after the addition of AI-2, CAI-1, and AIs [56]. Similarly, the resistance of phages increased when synthetic AHLs were added to the QS-deficient strain *Vibrio anguillarum* [57]. In addition, the expression of ompK, the phage receptor, decreased with AHL production, and the absence of QS signaling systems impeded both the DNA degradation process regulated by this system and the acquisition of immunity [58]. This phenomenon was observed in *Burkholderia glumae* and *Pectobacterium atrosepticum* as well. Furthermore, the RhlI and LasI QS systems in *P. aeruginosa* PA14 were found to control CRISPR-Cas gene expression [59]. Interestingly, doubling the number of sensitive cells in the culture using penicillic acid enhanced *P. aeruginosa* phage sensitivity [60]. These findings are not particularly surprising when considering that the use of quorum quenching (QQ) molecules is a highly promising strategy [3]. Indeed, incorporating phage therapy alongside QQ molecules may enhance bacteriophage susceptibility. Furthermore, in multi-microbial cultures, disrupting the quorum sensing (QS) of one species led to a decrease in overall biomass. Consequently, it can be concluded that the combined use of QQ molecules and phage treatment is effective against various microbial infections [61].

### In vivo antivirulence activity of quorum quenchers

In recent years, there has been a growing interest in elucidating the in vivo antivirulence activity of quorum quenchers (QQs). To assess the significance of quorum sensing inhibitors (QSIs) and quorum quenching enzymes (QQEs) on microbial virulence and pathogenicity, researchers have developed various models. These models employ three main approaches, ranging from simple unicellular models to more intricate systems. The following section provides a summary of these approaches.

### Model of amoebal infection

A model of amoebal infection has been developed to investigate the interaction between pathogens and host immune responses. The immune system responds to amoebae antigens through lysosomal digestion and phagocytosis defense mechanisms, similar to the bacterial inhibition mechanism used by macrophages [62]. Leveraging this close relationship, amoebae have been employed as a model to study the synthesis of bacterial virulence factors [63]. The ability of amoebae to thrive in the presence of pathogenic bacteria has been a valuable tool for assessing bacterial pathogenicity [64]. This approach has been utilized across various bacterial species to examine the relationship between quorum quenching (QQ) molecules and virulence factors. Recently, a QQ enzyme was evaluated using the amoeba model, based on a well-established test involving *Dictyostelium discoideum* and *P. aeruginosa*. In the plate-killing test of *D. discoideum*, the overproduction of *P. aeruginosa* PA14 with the aliphatic amidase disrupted quorum sensing (QS) and reduced pathogenicity toward the amoeba [63]. While this approach is quick and practical for studies, its application is limited due to the potential impact of amoeba species and culture conditions on result accuracy [65].

### **Infection model of*Caenorhabditis elegans***

*Caenorhabditis elegans* has become a predominant model for studying bacterial pathogenicity, offering valuable insights into the impact of quorum sensing (QS) on bacterial virulence [66]. Unlike amoebae, *C. elegans* possesses an innate immune system that closely mimics the human immune response, making it a highly relevant model for investigating pathogenicity [66]. The survival rate of *C. elegans* is commonly measured using specific microorganisms of interest. Numerous experimental trials, particularly with bacterial mutants deficient in autoinducer (AI) production, have been conducted to assess the significance of QS in pathogenicity. The introduction of quorum sensing inhibitors (QSIs) resulted in a reduction in worm mortality after infection with various *P. aeruginosa* strains, highlighting the role of QS in bacterial pathogenicity [67]. *C. elegans* has also been employed to explore the relationship between QS and pathogenicity in other Gram-negative bacteria, such as *Yersinia pseudotuberculosis* and *E. coli* [68]. Additionally, for Gram-positive bacteria, a connection between QS and pathogenicity has been established, as seen in *S. aureus* and *Enterococcus faecalis* [69]. In summary, these studies collectively demonstrate that QSI can effectively eliminate pathogenicity across a broad spectrum of bacteria.

*C. elegans* has been employed in vitro to validate the effectiveness of quorum sensing inhibitors (QSIs) and quorum quenching enzymes (QQEs). However, the impact on survival is subject to variation based on cultural conditions. For instance, the QQE BpiB09, which targets AHLs, significantly increased the survival of *C. elegans*, with rates reaching up to 100% [70]. Similarly, the quorum sensing inhibitor (QSI) 4-nitro-pyridine-N-oxide restored the worm’s survival completely by targeting the quorum sensing signaling system of P. aeruginosa PAO1 [15]. Natural quorum sensing inhibitor (QSI) extracts from *Callistemon viminalis*, *Bucida buceras*, and *Conocarpus* were shown to restore up to 87% survival [71]. Additionally, hamamelitannin and baicalin, known QSIs, have demonstrated synergistic effects with antibiotics [49].

*C. elegans* stands out as an especially useful invertebrate model, allowing for virtual screening of quorum quenching (QQ) compounds’ activity and studying bacterial mutations. It offers a comprehensive understanding of the regulation and modification of virulence factors by QQ [72]. QQEs and QSIs have proven effective in reducing mortality in *C. elegans* caused by a wide range of bacteria, showcasing the potential of QQ as an antipathogenic drug in multicellular organisms. However, certain restrictions exist, such as the living parameters of the worm, which differ from those of bacteria (e.g., the optimal growing temperature is 20 °C), and the significant physiological differences between roundworms and humans. Furthermore, like amoebae, various studies have highlighted the impact of conditions on assay results [73].

### Model of murine infection

To investigate the role of quorum sensing (QS) in bacterial infections, mammalian models such as mice or rats are commonly employed. Notably, deletions or mutations of QS-related genes have been shown to significantly reduce mortality and the severity of infections, including burn wounds [74], the prostate [75], and peritonitis [69] **(**Fig. 5).

QQ molecules have demonstrated a capacity to reduce mortality and hasten recovery. For instance, the furanone compound decreased *P. aeruginosa* colonization and associated mortality in lung infections of mammalian models [76]. Similarly, garlic extract [77] yielded comparable results in reducing *P. aeruginosa* colonization. QQEs have also proven effective in *P. aeruginosa* burn infections [45] and tea polyphenol quorum sensing inhibitors (QSIs) demonstrated efficacy in injury models [78]. Likewise, a variety of QSIs inhibited the expression of virulence factors in *S. aureus* during skin wound infections [79].

Moreover, extensive in vivo studies have explored the effectiveness of combining antibiotics with quorum quenching (QQ) molecules against both Gram-negative and Gram-positive bacteria. For instance, the combination of tobramycin and baicalin, a quorum sensing inhibitor (QSI), significantly reduced lung colonization by *B. cenocepacia* [49]. In another example, the combination of ciprofloxacin with a lactonase enzyme reduced mortality and bacterial spread to internal organs in mice with *P. aeruginosa* burn infections [45]. Additionally, the use of QSIs in combination with antibiotics markedly decreased microbial colonization induced by both *S. aureus* [80] and *S. epidermidis* [81].

These instances have generated interest in exploring the use of quorum quenching (QQ) molecules to mitigate antibiotic resistance in organ infections. Murine models prove invaluable for studying the impact of QQ molecules on bacterial infections, given their proximity to human physiology and their possessing both innate and adaptive immune systems. Additionally, murine models are often essential as preliminary tests before advancing to human clinical trials. The application of QQ molecules appears highly effective in reducing morbidity and adverse effects across various types of infections. However, murine models are less amenable to screening procedures compared to amoeba and C. elegans models, primarily due to ethical and practical considerations [82].

### Human clinical trials with quorum sensing inhibitors

The application of quorum quenching (QQ) molecules is rapidly advancing, with new inhibitors continually being discovered. Despite this progress, clinical applications are still under investigation, and only three human clinical trials on quorum sensing inhibitors (QSIs) have been conducted. The first trial utilized sub-inhibitory concentrations of the azithromycin antibiotic in the treatment of cystic fibrosis [83]. It demonstrated efficacy in vitro by inhibiting the signaling system in *P. aeruginosa*. Subsequently, it underwent evaluation in patients with ventilator-associated pneumonia [84]. Azithromycin’s antivirulence properties were examined in a high-risk patient group, but the findings did not reach statistical significance. The second study focused on garlic, a well-known quorum sensing inhibitor (QSI) utilized in cystic fibrosis treatment, although there is limited evidence supporting its therapeutic effect on pathogenic infections [85]. Finally, 5-fluorouracil (5-FU), an anti-cancer medicine, was discovered to inhibit virulence factors in vitro in *P. aeruginosa* and was applied as a coating for catheters. It demonstrated high efficacy in clinical trials [86]. Nonetheless, further research is required to confirm the therapeutic efficacy of this method in broader clinical trials.

### Use of quorum quenching molecules in medical devices

Medical equipment has been implicated in various hospital-acquired infections (HAIs) [87]. These infections, often caused by multidrug-resistant (MDR) and/or biofilm-forming bacteria, pose significant medical challenges and carry a high risk of mortality. Recognizing the potential of quorum quenching (QQ) to eliminate bacterial pathogenicity, novel medical devices incorporating QQ agents have been developed. Examples include the latest generations of contact lenses [88], implantable devices [89], catheters [90], or aerosols [91]. The initial application of quorum sensing inhibitors (QSIs) was in the modification of catheter surfaces. For instance, furanone was shown to reduce the biofilm of *S. epidermidis*, preventing infection for two months [92]. Similarly, catheters coated with 5-fluorouracil (5-FU) reduced Gram-negative bacterial contamination levels compared to conventional coatings. A varnish-based delivery method, producing the QSI thiazolidinedione-8, demonstrated efficacy against biofilms on catheters [93]. Combinations of furanone derivatives and dihydropyrrol-2-ones on glass surfaces decreased the adherence of both *P. aeruginosa* PAO1 and *S. aureus* SA38 [94].

Surfaces were also coated with anti-QS peptides such as TrAIP-II and AIP-I which were effective against *S. aureus* [95]. The combination of the daptomycin antibiotic with the QS-inhibiting peptide FS3 coated prosthesis demonstrated a synergistic effect against *staphylococcus* infection [96].

Likewise, the addition of the RNAIII inhibiting peptide (RIP) to the Dacron graft demonstrated high efficacy in reducing *S. epidermidis* infection [81]. In a rabbit model, the combination of α-amylase with acylase from *Bacillus amyloliquefaciens* resulted in decreased biofilm growth for both *E. coli* and *P. aeruginosa* [97]. Additionally, porcine kidney acylase adsorbed on nanofibers was utilized to create biocatalysts that inhibited *P. aeruginosa* PAO1 biofilm formation. The topical application of lactonase from *Bacillus* sp. ZA12 to mice infected with *P. aeruginosa* PAO1 had a synergistic effect with ciprofloxacin [45]. Considering the significance of enzyme stability in developing bio-based products, extremophile catalysts were also explored. The PLL SsoPox enzyme, isolated from *Sulfolobus solfataricus*, emerged as a particularly promising method for quenching bacterial pathogenicity [30]. This exceptionally durable enzyme was initially immobilized into nanoalumina membranes, maintaining its high efficiency in reducing virulence factor secretions of *P. aeruginosa* PAO1, such as pyocyanin and elastase [98]. After immobilization in a polyurethane covering using glutaraldehyde crosslinking, the SsoPox-W263I enzyme demonstrated its ability to reduce the pathogenicity of 51 *P. aeruginosa* clinical isolates from diabetic foot ulcers and maintained effectiveness toward *P. aeruginosa* PAO1 [99]. In a rat model with chronic respiratory infection by *P. aeruginosa* PAO1, the use of this mutant enzyme resulted in an increased survival rate [91]. Devices based on quorum quenching (QQ) have garnered significant attention due to their potential to prevent hospital-acquired infections (HAIs) by reducing bacterial pathogenicity and inhibiting biofilm formation. However, further in vivo and clinical trials are essential to establish their efficacy. The effectiveness of these devices must be validated across different bacterial strain models, including phenotypically and genetically diverse clinical isolates. While medical technology advanced devices face less regulation than pharmaceuticals, there is still ample room for innovation. Concerns must be addressed to validate the potential application of therapeutic procedures in the project. Nonetheless, the broad scope of quorum quenching enzymes (QQEs) and quorum sensing inhibitors (QSIs), along with numerous examples of potential clinical relevance, paves the way for the development of new devices.

## Conclusions and future perspectives

Over the past two decades, numerous studies have demonstrated the significant anti-pathogenic effects of QQ compounds and methods against a broad spectrum of bacteria. Various techniques for functionalizing medical equipment have shown substantial benefits. Yet, the probability of bacteria developing resistance to QQ mechanisms remains poorly understood. Resistance is the consequence of a natural evolutionary process that favors the formation of new, resistant strains. This is concerning because antibiotics significantly suppress the growth of sensitive bacteria. While some QSIs strongly inhibit growth, while others have minimal effects. QSI-resistant bacterial strains may likely develop, albeit at a slower rate than antibiotic-resistant strains. The specific QSI molecules and its impact on bacterial growth determine the emergence rate. Laboratory experiments have reported QSI-resistant bacteria, but the competitive advantage of these strains over QSI-sensitive ones remains unclear.

To mitigate QQ resistance, careful selection of QSI molecules is crucial, considering that many QSIs are toxic and require cell penetration for activation. The QQEs with potent virulence-inhibiting effects, may be promising candidates. Further studies employing QQEs are needed to identify putative QQ resistance mechanisms. The significant impact of QS on bacterial physiology suggests that QQ could not only eliminate bacterial pathogenicity but also restore antibiotic tolerance, paving the way for future therapeutic strategies. Surprisingly, disrupting bacterial signaling systems has consequences beyond the physiology of individual cells. More research is needed to understand the effects of QQ methods at both the individual bacterial and community levels. The next decade is expected to witness a significant increase in understanding QQ molecules, with widespread applications as coating agents in medical devices, potentially replacing bacteriostatic and bactericidal antibiotics. Additionally, innovative applications for QSIs include their use in combination therapies with antibiotics and phage therapy to enhance bacterial eradication, integration into agricultural practices for crop protection and livestock health, incorporation into medical device coatings to prevent biofilm formation, wound care treatments, food preservation, water treatment facilities, bioremediation efforts, engineered probiotics, oral care products, veterinary medicine, and anti-cancer strategies. These diverse applications underscore the potential of QSIs to address various challenges across multiple fields, promoting more effective and sustainable solutions. More investigations into the use of QQ molecules in human trials are anticipated.

## Data Availability

No datasets were generated or analysed during the current study.

## References

[1] El-Metwally MM, Mekawey AAI, El-Halmouch Y, Naga NG (2023) Symbiotic relationships with Fungi: from mutualism to Parasitism. Plant Mycobiome: diversity, interactions and uses. Springer, pp 375–413

[2] Naga NG, El-Badan DE-S, Rateb HS et al (2021) Quorum Sensing Inhibiting Activity of Cefoperazone and its Metallic Derivatives on *Pseudomonas aeruginosa* Frontiers in Cellular and Infection Microbiology 945. 10.3389/fcimb.2021.716789 10.3389/fcimb.2021.716789 PMC8515130 34660340

[3] Naga NG, El-Badan DE, Ghanem KM, Shaaban MI (2023) It is the time for quorum sensing inhibition as alternative strategy of antimicrobial therapy. Cell Communication Signal 21:133. 10.1186/s12964-023-01154-9 10.1186/s12964-023-01154-9 PMC10265836 37316831

[4] Horinouchi S (1999) γ-Butyrolactones that control secondary metabolism and cell differentiation in *Streptomyces*. Cell-cell Signal Bacteria 193–207

[5] Fuqua WC, Winans SC, Greenberg EP (1994) Quorum sensing in bacteria: the LuxR-LuxI family of cell density-responsive transcriptional regulators. J Bacteriol 176:269–275. 10.1128/jb.176.2.269-275.1994 8288518 10.1128/jb.176.2.269-275.1994 PMC205046

[6] Tiaden A, Hilbi H (2012) α-Hydroxyketone synthesis and sensing by *Legionella* and *Vibrio*. Sensors 12:2899–2919. 10.3390/s120302899 22736983 10.3390/s120302899 PMC3376566

[7] Deng Y, Wu J, Tao F, Zhang L-H (2011) Listening to a new language: DSF-based quorum sensing in Gram-negative bacteria. Chem Rev 111:160–173. 10.1021/cr100354f 21166386 10.1021/cr100354f

[8] Miller MB, Skorupski K, Lenz DH et al (2002) Parallel quorum sensing systems converge to regulate virulence in *Vibrio cholerae*. Cell 110:303–314. 10.1016/s0092-8674(02)00829-2 12176318 10.1016/s0092-8674(02)00829-2

[9] Pesci EC, Pearson JP, Seed PC, Iglewski BH (1997) Regulation of las and rhl quorum sensing in *Pseudomonas aeruginosa*. J Bacteriol 179:3127–3132. 10.1128/jb.179.10.3127-3132.1997 9150205 10.1128/jb.179.10.3127-3132.1997 PMC179088

[10] Dubern J-F, Diggle SP (2008) Quorum sensing by 2-alkyl-4-quinolones in *Pseudomonas aeruginosa* and other bacterial species. Mol Biosyst 4:882–888. 10.1039/b803796p 18704225 10.1039/b803796p

[11] Chu P-L, Feng Y-M, Long Z-Q et al (2023) Novel benzothiazole derivatives as potential anti-quorum sensing agents for managing plant bacterial diseases: synthesis, antibacterial activity assessment, and SAR study. J Agric Food Chem 71:6525–6540. 10.1021/acs.jafc.2c07810 37073686 10.1021/acs.jafc.2c07810

[12] Tang K, Zhang X-H (2014) Quorum quenching agents: resources for antivirulence therapy. Mar Drugs 12:3245–3282. 10.3390/md12063245 24886865 10.3390/md12063245 PMC4071575

[13] Park J, Jagasia R, Kaufmann GF et al (2007) Infection control by antibody disruption of bacterial quorum sensing signaling. Chem Biol 14:1119–1127. 10.1016/j.chembiol.2007.08.013 17961824 10.1016/j.chembiol.2007.08.013 PMC2088803

[14] Fetzner S (2015) Quorum quenching enzymes. J Biotechnol 201:2–14. 10.1016/j.jbiotec.2014.09.001 25220028 10.1016/j.jbiotec.2014.09.001

[15] Rasmussen TB, Bjarnsholt T, Skindersoe ME et al (2005) Screening for quorum-sensing inhibitors (QSI) by use of a novel genetic system, the QSI selector. J Bacteriol 187:1799–1814. 10.1128/JB.187.5.1799-1814.2005 15716452 10.1128/JB.187.5.1799-1814.2005 PMC1063990

[16] Naga NG, Zaki AA, El-Badan DE et al (2022) Methoxyisoflavan derivative from *Trigonella Stellata* inhibited quorum sensing and virulence factors of *Pseudomonas aeruginosa*. World J Microbiol Biotechnol 38:1–13. 10.1007/s11274-022-03337-x 10.1007/s11274-022-03337-x PMC9262770 35798919

[17] El-Mowafy SA, Abd El Galil KH, El-Messery SM, Shaaban MI (2014) Aspirin is an efficient inhibitor of quorum sensing, virulence and toxins in *Pseudomonas aeruginosa*. Microb Pathog 74:25–32. 10.1016/j.micpath.2014.07.008 25088031 10.1016/j.micpath.2014.07.008

[18] El-Mowafy SA, Abd El Galil KH, Habib E-SE, Shaaban MI (2017) Quorum sensing inhibitory activity of sub-inhibitory concentrations of β-lactams. Afr Health Sci 17:199–207. 10.4314/ahs.v17i1.25 29026394 10.4314/ahs.v17i1.25 PMC5636258

[19] Nalca Y, Jänsch L, Bredenbruch F et al (2006) Quorum-sensing antagonistic activities of azithromycin in *Pseudomonas aeruginosa* PAO1: a global approach. Antimicrobial agents and chemotherapy 50:1680–1688. 10.1128/AAC.50.5.1680-1688.2006. 10.1128/AAC.50.5.1680-1688.2006 PMC1472232 16641435

[20] Gabr MT, El-Gohary NS, El-Bendary ER, (2015) Synthesis, antimicrobial, antiquorum-sensing and cytotoxic activities of new series of benzothiazole derivatives. Chinese Chemical Letters 26:1522–1528

[21] Lin Y, Xu J, Hu J et al (2003) Acyl-homoserine lactone acylase from *Ralstonia* strain XJ12B represents a novel and potent class of quorum‐quenching enzymes. Mol Microbiol 47:849–860. 10.1046/j.1365-2958.2003.03351.x 12535081 10.1046/j.1365-2958.2003.03351.x

[22] Czajkowski R, Krzyżanowska D, Karczewska J et al (2011) Inactivation of AHLs by *Ochrobactrum* sp. A44 depends on the activity of a novel class of AHL acylase. Environ Microbiol Rep 3:59–68. 10.1111/j.1758-2229.2010.00188.x 23761232 10.1111/j.1758-2229.2010.00188.x

[23] Huang JJ, Petersen A, Whiteley M, Leadbetter JR (2006) Identification of QuiP, the product of gene PA1032, as the second acyl-homoserine lactone acylase of *Pseudomonas aeruginosa* PAO1. Appl Environ Microbiol 72:1190–1197. 10.1128/AEM.72.2.1190-1197.2006 16461666 10.1128/AEM.72.2.1190-1197.2006 PMC1392938

[24] Park S-Y, Kang H-O, Jang H-S et al (2005) Identification of extracellular N-acylhomoserine lactone acylase from a *Streptomyces* sp. and its application to quorum quenching. Appl Environ Microbiol 71:2632–2641. 10.1128/AEM.71.5.2632-2641.2005 15870355 10.1128/AEM.71.5.2632-2641.2005 PMC1087586

[25] Dong Y-H, Wang L-H, Xu J-L et al (2001) Quenching quorum-sensing-dependent bacterial infection by an N-acyl homoserine lactonase. Nature 411:813–817. 10.1038/35081101 11459062 10.1038/35081101

[26] Park S-Y, Lee SJ, Oh T-K et al (2003) AhlD, an N-acylhomoserine lactonase in *Arthrobacter* sp., and predicted homologues in other bacteria. Microbiology 149:1541–1550. 10.1099/mic.0.26269-0 12777494 10.1099/mic.0.26269-0

[27] Uroz S, Heinonsalo J (2008) Degradation of N-acyl homoserine lactone quorum sensing signal molecules by forest root-associated fungi. FEMS Microbiol Ecol 65:271–278. 10.1111/j.1574-6941.2008.00477.x 18400006 10.1111/j.1574-6941.2008.00477.x

[28] Camps J, Pujol I, Ballester F et al (2011) Paraoxonases as potential antibiofilm agents: their relationship with quorum-sensing signals in Gram-negative bacteria. Antimicrob Agents Chemother 55:1325–1331. 10.1128/AAC.01502-10 21199929 10.1128/AAC.01502-10 PMC3067127

[29] Faisal AJ, Said LA, Ali MR (2021) Quorum quenching effect of recombinant Paraoxonase-1 enzyme against Quorum sensing genes produced from *Pseudomonas aeruginosa*. Gene Rep 101412DOI. 10.1016/j.genrep.2021.101412

[30] Bzdrenga J, Daudé D, Remy B et al (2017) Biotechnological applications of quorum quenching enzymes. Chemico-Biol Interact 267:104–115. 10.1016/j.cbi.2016.05.028 10.1016/j.cbi.2016.05.028 27223408

[31] Shirazi J, Ain Q, Khan SJ et al (2021) Targeting Acyl Homoserine lactones (AHLs) by the Quorum quenching bacterial strains to Control Biofilm formation in *Pseudomonas Aeruginosa*. Saudi J Biol Sci. 10.1016/j.sjbs.2021.10.064 10.1016/j.sjbs.2021.10.064 PMC8913397 35280554

[32] Wang N, Jian W, Liang H et al (2024) Engineering a biomimicking strategy for discovering nonivamide-based quorum-sensing inhibitors for controlling bacterial infection. Eur J Med Chem 275:116609. 10.1016/j.ejmech.2024.116609 38896993 10.1016/j.ejmech.2024.116609

[33] Möker N, Dean CR, Tao J (2010) *Pseudomonas aeruginosa* increases formation of multidrug-tolerant persister cells in response to quorum-sensing signaling molecules. J Bacteriol 192:1946–1955. 10.1128/jb.01231-09 20097861 10.1128/JB.01231-09 PMC2838031

[34] Maura D, Hazan R, Kitao T et al (2016) Evidence for direct control of virulence and defense gene circuits by the *Pseudomonas aeruginosa* quorum sensing regulator. MvfR Sci Rep 6:1–14. 10.1038/srep34083 10.1038/srep34083 PMC5039717 27678057

[35] Liang H, Deng X, Li X et al (2014) Molecular mechanisms of master regulator VqsM mediating quorum-sensing and antibiotic resistance in *Pseudomonas aeruginosa*. Nucleic Acids Res 42:10307–10320. 10.1093/nar/gku586 25034696 10.1093/nar/gku586 PMC4176358

[36] Høiby N, Ciofu O, Johansen HK et al (2011) The clinical impact of bacterial biofilms. Int J Oral Sci 3:55–65. 10.4248/IJOS11026 21485309 10.4248/IJOS11026 PMC3469878

[37] Syafiuddin A, Boopathy R, Mehmood MA (2021) Recent advances on bacterial quorum quenching as an effective strategy to control biofouling in membrane bioreactors. Bioresource Technol Rep 100745. 10.1016/j.biteb.2021.100745

[38] Otto M (2006) Bacterial evasion of antimicrobial peptides by biofilm formation. Antimicrob Peptides Hum Disease 251–258. 10.1007/3-540-29916-5_10 10.1007/3-540-29916-5_10 16909925

[39] Qi P, Zhang T, Yang Y et al (2024) Beyond the β-amino alcohols framework: identification of novel β‐hydroxy pyridinium salt‐decorated pterostilbene derivatives as bacterial virulence factor inhibitors. Pest Manag Sci. 10.1002/ps.8116 38578108 10.1002/ps.8116

[40] Qi P-Y, Zhang T-H, Wang N et al (2023) Natural products-based botanical bactericides discovery: novel abietic acid derivatives as anti-virulence agents for plant disease management. J Agric Food Chem 71:5463–5475. 10.1021/acs.jafc.2c08392 37012216 10.1021/acs.jafc.2c08392

[41] González JF, Hahn MM, Gunn JS (2018) Chronic biofilm-based infections: skewing of the immune response. Pathogens Disease 76:fty023. 10.1093/femspd/fty023 29718176 10.1093/femspd/fty023 PMC6251518

[42] Michaelis C, Grohmann E (2023) Horizontal gene transfer of antibiotic resistance genes in biofilms. Antibiotics 12:328. 10.3390/antibiotics12020328 36830238 10.3390/antibiotics12020328 PMC9952180

[43] Zafer MM, Mohamed GA, Ibrahim SRM et al (2024) Biofilm-mediated infections by multidrug-resistant microbes: a comprehensive exploration and forward perspectives. Arch Microbiol 206:101. 10.1007/s00203-023-03826-z 38353831 10.1007/s00203-023-03826-z PMC10867068

[44] Zhao A, Sun J, Liu Y (2023) Understanding bacterial biofilms: from definition to treatment strategies. Front Cell Infect Microbiol 13:1137947. 10.3389/fcimb.2023.1137947 37091673 10.3389/fcimb.2023.1137947 PMC10117668

[45] Gupta P, Chhibber S, Harjai K (2015) Efficacy of purified lactonase and ciprofloxacin in preventing systemic spread of *Pseudomonas aeruginosa* in murine burn wound model. Burns 41:153–162. 10.1016/j.burns.2014.06.009 25015706 10.1016/j.burns.2014.06.009

[46] Olsen I (2015) Biofilm-specific antibiotic tolerance and resistance. Eur J Clin Microbiol Infect Dis 34:877–886. 10.1007/s10096-015-2323-z 25630538 10.1007/s10096-015-2323-z

[47] Jakobsen TH, Bjarnsholt T, Jensen PØ et al (2013) Targeting quorum sensing in *Pseudomonas aeruginosa* biofilms: current and emerging inhibitors. Future Microbiol 8:901–921. 10.2217/fmb.13.57 23841636 10.2217/fmb.13.57

[48] Maura D, Rahme LG (2017) Pharmacological inhibition of the *Pseudomonas aeruginosa* MvfR quorum-sensing system interferes with biofilm formation and potentiates antibiotic-mediated biofilm disruption. Antimicrobial agents and chemotherapy 61. 10.1128/AAC.01362-17 10.1128/AAC.01362-17 PMC5700327 28923875

[49] Brackman G, Cos P, Maes L et al (2011) Quorum sensing inhibitors increase the susceptibility of bacterial biofilms to antibiotics in vitro and in vivo. Antimicrob Agents Chemother 55:2655–2661. 10.1128/AAC.00045-11 21422204 10.1128/AAC.00045-11 PMC3101409

[50] Guo Q, Wei Y, Xia B et al (2016) Identification of a small molecule that simultaneously suppresses virulence and antibiotic resistance of *Pseudomonas aeruginosa*. Sci Rep 6:1–15. 10.1038/srep19141 26751736 10.1038/srep19141 PMC4707474

[51] Brssow H, Hendrix RW (2002) Phage genomics. Cell 108:637. 10.1016/s0092-8674(01)00637-7 11792317 10.1016/s0092-8674(01)00637-7

[52] Labrie SJ, Samson JE, Moineau S (2010) Bacteriophage resistance mechanisms. Nat Rev Microbiol 8:317–327. 10.1038/nrmicro2315 20348932 10.1038/nrmicro2315

[53] Chapman-McQuiston E, Wu XL (2008) Stochastic receptor expression allows sensitive bacteria to evade phage attack. Part I: experiments. Biophys J 94:4525–4536. 10.1529/biophysj.107.120212 18310238 10.1529/biophysj.107.120212 PMC2480656

[54] Barrangou R, Fremaux C, Deveau H et al (2007) CRISPR provides acquired resistance against viruses in prokaryotes. Science 315:1709–1712. 10.1126/science.1138140 17379808 10.1126/science.1138140

[55] Glessner A, Smith RS, Iglewski BH, Robinson JB (1999) Roles of *Pseudomonas aeruginosa* Las and Rhl quorum-sensing systems in control of twitching motility. J Bacteriol 181:1623–1629. 10.1128/JB.181.5.1623-1629.1999 10049396 10.1128/jb.181.5.1623-1629.1999 PMC93554

[56] Hoque M, Mozammel IB, Naser SMN, Bari J, Zhu JJ, Mekalanos and Shah M. Faruque. 2016. Quorum Regulated Resistance of *Vibrio cholerae* against Environmental bacteriophages. Sci Rep 6:37956. 10.1038/srep37956 10.1038/srep37956 PMC5124996 27892495

[57] Tan D, Svenningsen S (2015) Lo; Middelboe, M. Quorum sensing determines the choice of antiphage defense strategy in. Vibrio anguillarum MBio 6:e00627. 10.1128/mBio.00627-15 26081633 10.1128/mBio.00627-15 PMC4471561

[58] Gao R, Krysciak D, Petersen K et al (2015) Genome-wide RNA sequencing analysis of quorum sensing-controlled regulons in the plant-associated *Burkholderia glumae* PG1 strain. Appl Environ Microbiol 81:7993–8007. 10.1128/AEM.01043-15 26362987 10.1128/AEM.01043-15 PMC4651095

[59] Høyland-Kroghsbo NM, Paczkowski J, Mukherjee S et al (2017) Quorum sensing controls the Pseudomonas aeruginosa CRISPR-Cas adaptive immune system. Proc Natl Acad Sci 114:131–135 27849583 10.1073/pnas.1617415113 PMC5224376

[60] Qin X, Sun Q, Yang B et al (2017) Quorum sensing influences phage infection efficiency via affecting cell population and physiological state. J Basic Microbiol 57:162–170. 10.1073/pnas.1617415113 27714824 10.1002/jobm.201600510

[61] Mumford R, Friman V (2017) Bacterial competition and quorum-sensing signalling shape the eco‐evolutionary outcomes of model in vitro phage therapy. Evol Appl 10:161–169. 10.1111/eva.12435 28127392 10.1111/eva.12435 PMC5253424

[62] Hilbi H, Weber SS, Ragaz C et al (2007) Environmental predators as models for bacterial pathogenesis. Environ Microbiol 9:563–575. 10.1111/j.1462-2920.2007.01238.x 17298357 10.1111/j.1462-2920.2007.01238.x

[63] Clamens T, Rosay T, Crépin A et al (2017) The aliphatic amidase AmiE is involved in regulation of *Pseudomonas aeruginosa* virulence. Sci Rep 7:1–16. 10.1038/srep41178 28117457 10.1038/srep41178 PMC5259723

[64] Pukatzki S, Kessin RH, Mekalanos JJ (2002) The human pathogen Pseudomonas aeruginosa utilizes conserved virulence pathways to infect the social amoeba Dictyostelium Discoideum. Proc Natl Acad Sci 99:3159–3164. 10.1073/pnas.052704399 11867744 10.1073/pnas.052704399 PMC122489

[65] Weitere M, Bergfeld T, Rice SA et al (2005) Grazing resistance of *Pseudomonas aeruginosa* biofilms depends on type of protective mechanism, developmental stage and protozoan feeding mode. Environ Microbiol 7:1593–1601. 10.1111/j.1462-2920.2005.00851.x 16156732 10.1111/j.1462-2920.2005.00851.x

[66] Ermolaeva MA, Schumacher B (2014) Insights from the worm: the *C. Elegans* model for innate immunity. Seminars in immunology. Elsevier, pp 303–309. DOI: 10.1016/j.smim.2014.04.005 10.1016/j.smim.2014.04.005 PMC4248339 24856329

[67] Darby C, Cosma CL, Thomas JH, Manoil C (1999) Lethal paralysis of *Caenorhabditis elegans* by *Pseudomonas aeruginosa*. Proceedings of the National Academy of Sciences 96:15202–15207. 10.1073/pnas.96.26.15202 10.1073/pnas.96.26.15202 PMC24797 10611362

[68] Lee K-M, Lim J, Nam S et al (2011) Inhibitory effects of broccoli extract on Escherichia coli O157: H7 quorum sensing and in vivo virulence. FEMS Microbiol Lett 321:67–74. 10.1111/j.1574-6968.2011.02311.x 21592195 10.1111/j.1574-6968.2011.02311.x

[69] Sifri CD, Mylonakis E, Singh KV et al (2002) Virulence effect of *Enterococcus faecalis* protease genes and the quorum-sensing locus fsr in *Caenorhabditis elegans* and mice. Infect Immun 70:5647–5650. 10.1128/IAI.70.10.5647-5650.2002 12228293 10.1128/IAI.70.10.5647-5650.2002 PMC128331

[70] Bijtenhoorn P, Mayerhofer H, Müller-Dieckmann J et al (2011) A novel metagenomic short-chain dehydrogenase/reductase attenuates *Pseudomonas aeruginosa* biofilm formation and virulence on *Caenorhabditis elegans*. PLoS ONE 6:e26278. 10.1371/journal.pone.0026278 22046268 10.1371/journal.pone.0026278 PMC3202535

[71] Adonizio A, Leal SM Jr, Ausubel FM, Mathee K (2008) Attenuation of *Pseudomonas aeruginosa* virulence by medicinal plants in a *Caenorhabditis elegans* model system. J Med Microbiol 57:809–813. 10.1099/jmm.0.47802-0 18566137 10.1099/jmm.0.47802-0

[72] Kong C, Eng S-A, Lim M-P, Nathan S (2016) Beyond traditional antimicrobials: A *Caenorhabditis elegans* model for discovery of novel anti-infectives. Frontiers in microbiology 7:1956. 10.3389/fmicb.2016.01956 10.3389/fmicb.2016.01956 PMC5133244 27994583

[73] Mahajan-Miklos S, Tan M-W, Rahme LG, Ausubel FM (1999) Molecular mechanisms of bacterial virulence elucidated using a *Pseudomonas aeruginosa*–*Caenorhabditis elegans* pathogenesis model. Cell 96:47–56. 10.1016/s0092-8674(00)80958-7 9989496 10.1016/s0092-8674(00)80958-7

[74] Tan M-W, Rahme LG, Sternberg JA et al (1999) Pseudomonas aeruginosa killing of Caenorhabditis elegans used to identify P. Aeruginosa virulence factors. Proc Natl Acad Sci 96:2408–2413. 10.1073/pnas.96.5.2408 10051655 10.1073/pnas.96.5.2408 PMC26797

[75] Nelson LK, D’Amours GH, Sproule-Willoughby KM et al (2009) *Pseudomonas aeruginosa* Las and Rhl quorum-sensing systems are important for infection and inflammation in a rat prostatitis model. Microbiology 155:2612–2619. 10.1099/mic.0.028464-0 19460822 10.1099/mic.0.028464-0

[76] Wu H, Song Z, Hentzer M et al (2004) Synthetic furanones inhibit quorum-sensing and enhance bacterial clearance in *Pseudomonas aeruginosa* lung infection in mice. J Antimicrob Chemother 53:1054–1061. 10.1093/jac/dkh223 15117922 10.1093/jac/dkh223

[77] Hoffmann N, Lee B, Hentzer M et al (2007) Azithromycin blocks quorum sensing and alginate polymer formation and increases the sensitivity to serum and stationary-growth-phase killing of *Pseudomonas aeruginosa* and attenuates chronic *P. aeruginosa* lung infection in Cftr(–/–)mice. Antimicrob Agents Chemother 51:3677–3687. 10.1128/AAC.01011-06 17620382 10.1128/AAC.01011-06 PMC2043275

[78] Yin H, Deng Y, Wang H et al (2015) Tea polyphenols as an antivirulence compound disrupt quorum-sensing regulated pathogenicity of *Pseudomonas aeruginosa*. Sci Rep 5:1–12. 10.1038/SREP16158 10.1038/srep16158 PMC4637895 26548447

[79] Muhs A, Lyles JT, Parlet CP et al (2017) Virulence inhibitors from Brazilian peppertree block quorum sensing and abate dermonecrosis in skin infection models. Sci Rep 7:1–15. 10.1038/srep42275 28186134 10.1038/srep42275 PMC5301492

[80] Simonetti O, Cirioni O, Cacciatore I et al (2016) Efficacy of the quorum sensing inhibitor FS10 alone and in combination with tigecycline in an animal model of staphylococcal infected wound. PLoS ONE 11:e0151956. 10.1371/journal.pone.0151956 27253706 10.1371/journal.pone.0151956 PMC4890846

[81] Balaban N, Giacometti A, Cirioni O et al (2003) Use of the quorum-sensing inhibitor RNAIII-inhibiting peptide to prevent biofilm formation in vivo by drug-resistant *Staphylococcus epidermidis*. J Infect Dis 187:625–630. 10.1086/345879 12599079 10.1086/345879

[82] Abdullahi A, Amini-Nik S, Jeschke MG (2014) Animal models in burn research. Cell Mol Life Sci 71:3241–3255. 10.1007/s00018-014-1612-5 24714880 10.1007/s00018-014-1612-5 PMC4134422

[83] Saiman L, Marshall BC, Mayer-Hamblett N et al (2003) Azithromycin in patients with cystic fibrosis chronically infected with *Pseudomonas aeruginosa*: a randomized controlled trial. JAMA 290:1749–1756. 10.1001/jama.290.13.1749 14519709 10.1001/jama.290.13.1749

[84] Tateda K, Comte R, Pechere J-C et al (2001) Azithromycin inhibits quorum sensing in *Pseudomonas aeruginosa*. Antimicrob Agents Chemother 45:1930–1933. 10.1128/AAC.45.6.1930-1933.2001 11353657 10.1128/AAC.45.6.1930-1933.2001 PMC90577

[85] Smyth AR, Cifelli PM, Ortori CA et al (2010) Garlic as an inhibitor of *Pseudomonas aeruginosa* quorum sensing in cystic fibrosis—a pilot randomized controlled trial. Pediatr Pulmonol 45:356–362. 10.1002/ppul.21193 20306535 10.1002/ppul.21193

[86] Walz JM, Avelar RL, Longtine KJ et al (2010) Anti-infective external coating of central venous catheters: a randomized, noninferiority trial comparing 5-fluorouracil with chlorhexidine/silver sulfadiazine in preventing catheter colonization. Crit Care Med 38:2095–2102. 10.1097/CCM.0b013e3181f265ba 20711070 10.1097/CCM.0b013e3181f265ba

[87] Neoh KG, Li M, Kang E-T et al (2017) Surface modification strategies for combating catheter-related complications: recent advances and challenges. J Mater Chem B 5:2045–2067. 10.1039/c6tb03280j 32263678 10.1039/c6tb03280j

[88] Jain N, Bhosale P, Tale V (2016) Biofilm formation on contact lenses by bacterial pathogens. J Pharm Res 10:50–53

[89] Francolini I, Vuotto C, Piozzi A, Donelli G (2017) Antifouling and antimicrobial biomaterials: an overview. Apmis 125:392–417. 10.1111/apm.12675 28407425 10.1111/apm.12675

[90] Mandakhalikar KD, Chua RR, Tambyah PA (2016) New technologies for prevention of catheter associated urinary tract infection. Curr Treat Options Infect Dis 8:24–41

[91] Hraiech S, Hiblot J, Lafleur J et al (2014) Inhaled lactonase reduces *Pseudomonas aeruginosa* quorum sensing and mortality in rat pneumonia. PLoS ONE 9:e107125. 10.1371/journal.pone.0107125 25350373 10.1371/journal.pone.0107125 PMC4211673

[92] Hume EBH, Baveja J, Muir B et al (2004) The control of S*taphylococcus epidermidis* biofilm formation and in vivo infection rates by covalently bound furanones. Biomaterials 25:5023–5030. 10.1016/j.biomaterials.2004.01.048 15109864 10.1016/j.biomaterials.2004.01.048

[93] Shenderovich J, Feldman M, Kirmayer D et al (2015) Local sustained-release delivery systems of the antibiofilm agent thiazolidinedione-8 for prevention of catheter-associated urinary tract infections. Int J Pharm 485:164–170. 10.1016/j.ijpharm.2015.02.067 25769292 10.1016/j.ijpharm.2015.02.067

[94] Taunk A, Ho KKK, Iskander G et al (2016) Surface immobilization of antibacterial quorum sensing inhibitors by photochemical activation. J Biotechnol Biomater 6:1000238. 10.4172/2155-952X.1000238

[95] Kim MK, Zhao A, Wang A et al (2017) Surface-attached molecules control *Staphylococcus aureus* quorum sensing and biofilm development. Nat Microbiol 2:1–12. 10.1038/nmicrobiol.2017.80 10.1038/nmicrobiol.2017.80 PMC5526357 28530651

[96] Cirioni O, Mocchegiani F, Cacciatore I et al (2013) Quorum sensing inhibitor FS3-coated vascular graft enhances daptomycin efficacy in a rat model of staphylococcal infection. Peptides 40:77–81. 10.1016/j.peptides.2012.12.002 23262356 10.1016/j.peptides.2012.12.002

[97] Ivanova K, Fernandes MM, Mendoza E, Tzanov T (2015) Enzyme multilayer coatings inhibit *Pseudomonas aeruginosa* biofilm formation on urinary catheters. Appl Microbiol Biotechnol 99:4373–4385. 10.1007/s00253-015-6378-7 25582561 10.1007/s00253-015-6378-7

[98] Ng FSW, Wright DM, Seah SYK (2011) Characterization of a phosphotriesterase-like lactonase from *Sulfolobus solfataricus* and its immobilization for disruption of quorum sensing. Appl Environ Microbiol 77:1181–1186. 10.1128/AEM.01642-10 21183649 10.1128/AEM.01642-10 PMC3067241

[99] Guendouze A, Plener L, Bzdrenga J et al (2017) Effect of quorum quenching lactonase in clinical isolates of *Pseudomonas aeruginosa* and comparison with quorum sensing inhibitors. Front Microbiol 8:227. 10.3389/fmicb.2017.00227 28261183 10.3389/fmicb.2017.00227 PMC5306132

